# Influenza Vaccine Effectiveness Against Influenza-like Illness in Post-Operative Children with Congenital Heart Disease: A Retrospective Cohort Study Using Inverse Probability of Treatment Weighting

**DOI:** 10.3390/vaccines14070631

**Published:** 2026-07-20

**Authors:** Rui Liu, Pengfei Deng, Tian Yang, Yun Pan, Zhuoming Xu, Laibao Yang, Caoyi Xue, Qizhang Wang

**Affiliations:** 1 School of Public Health, Dali University, 2 Hongsheng Road, Dali 671003, China; m13678783197@163.com; 2 Shanghai Pudong New Area Center for Disease Control and Prevention (Shanghai Pudong New Area Health Supervision Institute), 3039 Zhangyang Road, Pudong New Area, Shanghai 200136, China; masterdpf@163.com (P.D.); yy815201912@163.com (T.Y.); yanglaibao1985@163.com (L.Y.); 3 Department of Pediatric Cardiology, Shanghai Children’s Medical Center, Shanghai Jiao Tong University School of Medicine, 1678 Dongfang Road, Pudong New Area, Shanghai 200127, China; huhujascha@163.com (Y.P.); zmxcicu@163.com (Z.X.); 4 Pudong Institute for Health Development, 818 Laiyang Road, Pudong New Area, Shanghai 200129, China

**Keywords:** congenital heart disease, children, influenza vaccine, vaccine effectiveness, inverse probability of treatment weighting, doubly robust estimation

## Abstract

**Background**: Children with congenital heart disease (CHD) are highly vulnerable to influenza, yet real-world vaccine effectiveness (VE) data in this post-operative population are lacking, especially in China. This study estimated influenza VE against medically attended influenza-like illness (ILI) in post-operative children with CHD using inverse probability of treatment weighting (IPTW) to address confounding. **Methods**: We retrospectively enrolled 194 children who underwent CHD surgery in Shanghai (January–July 2023). Vaccination records were obtained from the Shanghai Immunization Information System, and ILI episodes were captured via structured interviews. The primary analysis used doubly robust quasi-Poisson regression on an IPTW pseudo-population. VE = (1 − RR) × 100%. Sensitivity analyses included multivariable Poisson regression, marginal structural models, and stabilized IPTW. **Results**: Vaccination coverage was 21.6% (42/194). After IPTW, all covariates were well-balanced (SMD < 0.10). The primary VE was 50.9% (95% CI: 11.0–72.9%). Sensitivity estimates ranged from 49.6% to 56.6%. Subgroup analyses showed consistently protective point estimates. **Conclusions**: Influenza vaccination provided moderate, robust protection (≈51% VE) against ILI in post-operative CHD children, comparable to healthy pediatric populations. Our results reinforce the urgent need to prioritize influenza immunization in this vulnerable group and offer critical baseline data for China.

## 1. Introduction

Seasonal influenza causes an estimated 3 to 5 million severe cases and 290,000 to 650,000 respiratory-related deaths annually [1,2]. Infants, young children, and individuals with chronic cardiopulmonary conditions are at increased risk of severe morbidity and mortality. Children with congenital heart disease (CHD) are among the most vulnerable of these groups. CHD is the most prevalent birth defect in China, affecting approximately 8 per 1000 live births, which corresponds to roughly 150,000 new cases each year [3]. Children with CHD not only have a higher susceptibility to influenza infection but also face a greater likelihood of severe complications. In a U.S. national database study, 4.3% of children hospitalized for influenza had an underlying CHD diagnosis [4]. Compared with healthy peers, children with CHD hospitalized for influenza experience higher rates of in-hospital mortality, acute respiratory failure, mechanical ventilation, and prolonged hospitalization [5]. Post-operative children with CHD represent a particularly challenging subgroup for influenza prevention, as their recovery period and immunological status may influence both vaccine uptake and response. This increased risk is linked to residual hemodynamic abnormalities, systemic inflammation following cardiopulmonary bypass, and, in some cases, prolonged use of immunomodulatory or cardiac medications [6].

Despite these risks, influenza vaccination coverage among children with CHD in China remains low. National guidelines recommend annual influenza vaccination for children aged ≥6 months, prioritizing those with chronic conditions. Expert consensus has also indicated that CHD should not be considered a contraindication to vaccination. In practice, however, the influenza vaccine is classified as a non-National Immunization Program vaccine and administered on a voluntary basis. A recent big-data study from Yinzhou District reported that although 86.74% of children with CHD had received at least one dose of National Immunization Program vaccines, coverage for non-NIP vaccines, such as the influenza vaccine, was only 7.74% [7]. Improving vaccination coverage requires evidence of meaningful vaccine protection, a gap that local VE estimates can help fill. To date, however, no real-world VE data are available for post-operative children with CHD in China, and the global evidence base for this population remains limited [8]. Most existing VE studies have been conducted in Western settings and have largely relied on hospitalized influenza cases, whereas data on medically attended ILI in community settings are sparse for this group. According to the Chinese National Influenza Surveillance Network, the 2024–2025 influenza season was characterized by co-circulation of influenza A(H1N1)pdm09 and A(H3N2) as the predominant subtypes, with most circulating strains antigenically similar to the corresponding vaccine components [9]. This provided a favorable context for evaluating VE. To address this evidence gap, we conducted a retrospective cohort study to estimate real-world VE against medically attended ILI in post-operative children with CHD in Shanghai. We hypothesized that influenza vaccination confers moderate protection against ILI in this cohort. Inverse probability of treatment weighting (IPTW) was used to control for confounding by indication, supplemented by doubly robust estimation and sensitivity analyses to ensure the robustness of the findings.

## 2. Materials and Methods

### 2.1. Study Design and Population

This retrospective cohort study was conducted at a tertiary children’s hospital in Shanghai, China. The study consecutively enrolled children who underwent cardiac surgery for CHD between 1 January and 31 July 2023.

The inclusion criteria were defined as follows: (1) aged 0–17 years at the time of surgery; (2) a confirmed CHD diagnosis via echocardiography and/or cardiac catheterization; (3)clinical documentation of hemodynamic stability and active post-surgical recovery; (4) no history of immunosuppressive agent usage or blood transfusions within the preceding 3 months; and (5) expressed willingness by guardians to participate in long-term follow-up.

The exclusion criteria comprised: (1) the presence of active co-existing infectious diseases at the time of enrollment; (2) any documented comorbidities known to compromise immune function (e.g., primary immunodeficiencies or HIV); (3) a history of severe allergic diseases that could potentially impact vaccination status; and (4) known contraindications to influenza vaccination, including a history of severe allergic reactions to any vaccine component.

### 2.2. Data Collection and Outcome Definition

Data collection was executed in two structured phases. The initial telephone interview, conducted in August 2024, collected information on baseline ILI history during the pre-study season (1 August 2023–31 July 2024), demographic profiles, and established risk factors for respiratory infections. The subsequent interview, completed in December 2025, recorded ILI episodes occurring throughout the primary observation period (1 August 2024–31 December 2025).

All interviews were administered by uniformly trained and certified personnel who remained blinded to the study’s core hypothesis, utilizing a structured questionnaire on the Wenjuanxing platform (Changsha Ranxing Information Technology Co., Ltd., Changsha, China). To systematically mitigate recall bias, the following measures were implemented: (1) interviewers employed structured calendar-anchoring methodologies; (2) the elapsed time between the end of the season and the interview was strictly kept under 6 months for the 2024–2025 season; and (3) parents were explicitly encouraged to reference personal medical records during the interviews. Detailed influenza vaccination histories—encompassing vaccine types, dose counts, and administration dates—were retrieved from the Shanghai Immunization Planning Information System (SIPIS), a validated, population-based electronic health registry maintained by the Shanghai Municipal Center for Disease Control and Prevention (Shanghai, China). The primary exposure of interest was defined as the receipt of at least one dose of the influenza vaccine during the interval between the two follow-up interviews.

The primary clinical outcome, ILI, was classified according to World Health Organization (WHO) criteria: acute symptom onset (≤7 days), an axillary temperature of ≥38 °C, and the presence of at least two associated symptoms (chills, malaise, headache, myalgia/arthralgia, cough, sore throat, nasal congestion, or nasopharyngeal erythema). To optimize diagnostic specificity, the analysis was strictly restricted to medically attended ILI episodes. These were defined as events prompting a visit to a healthcare facility (community health center, pediatric outpatient clinic, or hospital) resulting in a formally documented clinical diagnosis of ILI or an acute respiratory infection in the patient’s medical records.

### 2.3. Statistical Analysis

Categorical variables are reported as counts and percentages (%), while continuous variables are expressed as either the mean ± standard deviation (SD) or the median with the interquartile range (IQR). Adhering to methodological recommendations for propensity score analyses, baseline comparability was assessed primarily using standardized mean differences (SMDs) rather than traditional *p*-values; an absolute SMD of <0.10 was established as the threshold indicative of negligible covariate imbalance [10,11].

Observational VE studies are inherently limited by confounding by indication—where systematic differences between vaccinated and unvaccinated groups concurrently influence the clinical outcome risk [12]. IPTW effectively addresses this by mathematically reweighting the observational cohort to generate a pseudo-population in which treatment assignment operates independently of measured covariates, thereby approximating the covariate exchangeability of a randomized trial [13,14]. A multivariable logistic regression model was utilized to calculate the propensity score (PS), designating vaccination status as the dependent variable. The model incorporated the following a priori selected covariates: sex, age (in months), diagnosis type (left-to-right shunt versus other anomalies), passive smoking exposure, prior ILI history, BMI Z-score classification (normal versus abnormal), and engagement in any preventive behaviors. This variable selection aligns closely with the recommendations of Brookhart et al. [15] and VanderWeele [16], prioritizing factors known to be associated with both the exposure and the clinical outcome. Average treatment effect (ATE) weights were subsequently computed (1/PS for the vaccinated cohort; 1/(1 − PS) for the unvaccinated cohort) and truncated at the 99th percentile to minimize the disproportionate influence of extreme statistical weights [17]. Covariate balance was verified via the absolute SMD.

Within this weighted sample, we employed a doubly robust quasi-Poisson regression model, incorporating robust (sandwich) standard errors. The doubly robust framework integrates all covariates from the PS model as additional adjustment variables in the outcome model, yielding consistent effect estimates provided that at least one of the underlying models (PS or outcome) is correctly specified [18,19]. VE was calculated as (1 − RR) × 100%, and this model was designated as the primary analysis.

Three pre-specified sensitivity analyses were executed to validate the robustness of the findings: (1) unweighted conventional multivariable Poisson regression; (2) a marginal structural model (representing IPTW without secondary outcome-model adjustments); and (3) IPTW utilizing stabilized weights [20]. Furthermore, subgroup analyses were stratified by sex, diagnosis type, passive smoking exposure, prior ILI history, BMI category, and preventive behaviors. For each distinct stratum, the PS model was independently re-estimated prior to the application of IPTW. Subgroups comprising fewer than 20 individuals or presenting confidence interval widths exceeding 200 percentage points were designated as purely exploratory. Due to limited statistical power, formal interaction tests were not performed.

All statistical analyses were executed using R version 4.5.2 (R Foundation for Statistical Computing, Vienna, Austria), utilizing the survey (version 4.5), dplyr (version 1.2.0), and ggplot2 (version 4.0.3) packages. A two-sided *p*-value of <0.05 was considered statistically significant.

## 3. Results

### 3.1. Study Population and Baseline Characteristics

**Figure 1:**
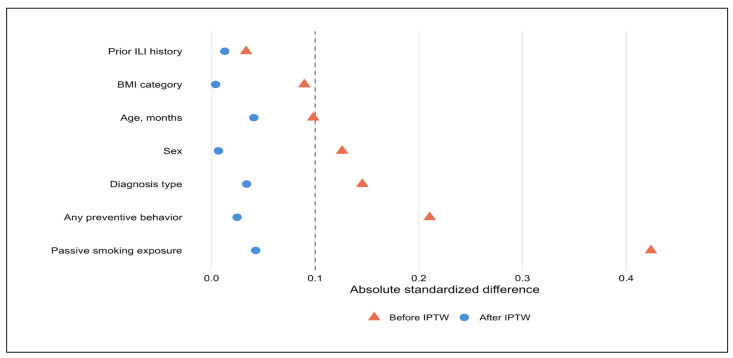
Covariate balance before and after inverse probability of treatment weighting. The dashed line indicates the prespecified threshold for adequate balance (SMD = 0.10).

Among the 194 post-operative children with CHD evaluated, 42 (21.6%) received ≥1 dose of the influenza vaccine during the follow-up period. This recorded coverage falls markedly short of the 75% target recommended by the World Health Organization for high-risk populations, including those with chronic medical conditions [21]. Prior to statistical weighting, notable imbalances were evident between the groups. Specifically, passive smoking exposure was substantially less frequent in the vaccinated cohort (7.1% compared to 21.7% in the unvaccinated group; absolute SMD = 0.424), with minor imbalances also observed concerning preventive behaviors (SMD = 0.210) and CHD diagnosis type (SMD = 0.146) (Table 1). Following the application of IPTW, optimal balance was achieved across all evaluated covariates. Absolute SMDs ranged narrowly from 0.004 to 0.043, successfully falling well below the strict 0.10 threshold. Crucially, the covariate displaying the largest pre-weighting imbalance—passive smoking exposure—was completely normalized (SMD reduced from 0.424 to 0.043) (Table 1; Figure 1).

### 3.2. Vaccine Effectiveness: Primary and Sensitivity Analyses

**Figure 2:**
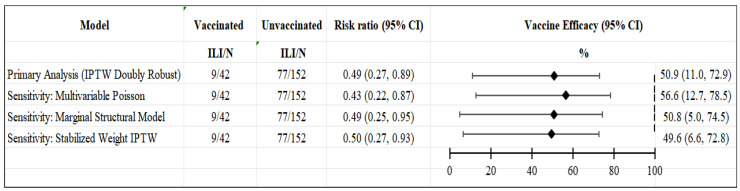
Influenza vaccine effectiveness against influenza-like illness: primary and sensitivity analyses. VE = (1 − RR) × 100%. All models were adjusted for sex, age, diagnosis type, passive smoking exposure, prior ILI history, BMI category, and any preventive behavior. The IPTW doubly robust model was designated as the primary analysis. Diamonds represent point estimates of VE; horizontal lines indicate 95% confidence intervals; the dashed vertical line indicates the null value (VE = 0%).

Under the primary doubly robust IPTW model, influenza vaccination demonstrated a 50.9% reduction in the risk of ILI (95% CI: 11.0–72.9%; risk ratio [RR] = 0.49, 95% CI: 0.27–0.89). Sensitivity analyses yielded highly concordant results, affirming the robustness of the primary model. The conventional multivariable Poisson regression estimated a VE of 56.6% (95% CI: 12.7–78.5%; RR = 0.43, 95% CI: 0.22–0.87). Similarly, the marginal structural model (unadjusted outcome-model IPTW) calculated a VE of 50.8% (95% CI: 5.0–74.5%; RR = 0.49, 95% CI: 0.25–0.95), and the stabilized IPTW approach yielded a VE of 49.6% (95% CI: 6.6–72.8%; RR = 0.50, 95% CI: 0.27–0.93). Across these four diverse analytical methodologies, VE point estimates remained remarkably stable between 49.6% and 56.6%, with all confidence intervals consistently excluding the statistical null (Figure 2).

### 3.3. Subgroup Analyses

**Figure 3:**
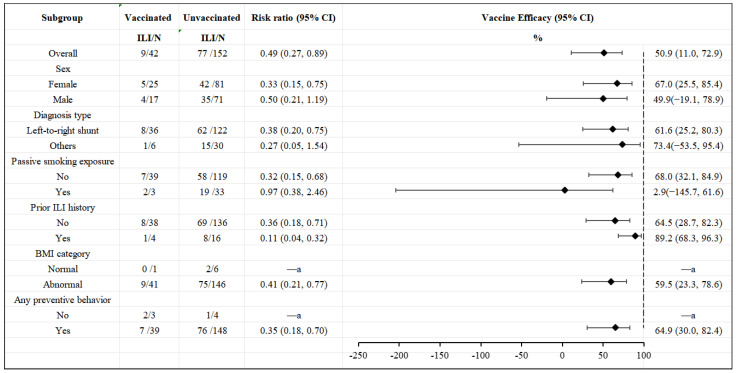
Subgroup analyses of influenza vaccine effectiveness against influenza-like illness using IPTW. VE = (1 − RR) × 100%. Propensity scores were re-estimated independently within each subgroup. Estimates marked with “a” are considered exploratory due to small sample size (n < 25) or wide confidence intervals. Diamonds represent point estimates of VE; horizontal lines indicate 95% confidence intervals; the dashed vertical line indicates the null value (VE = 0%).

Subgroup analyses, with propensity scores re-estimated within each stratum (Figure 3), showed generally consistent protective effects across most subgroups, although estimates were imprecise in smaller strata. Notably, VE appeared higher in females and among children without passive smoke exposure, while the protective effect was substantially attenuated in those exposed to passive smoking. Estimates for subgroups with very small sample sizes were exploratory and should be interpreted with caution.

## 4. Discussion

In this retrospective investigation encompassing 194 post-operative children with CHD in Shanghai, influenza vaccination was associated with a 51% decline in the risk of contracting ILI (95% CI: 11.0–72.9%). This protective effect demonstrated remarkable consistency across four distinct analytical frameworks—doubly robust IPTW, conventional multivariable Poisson regression, marginal structural models, and stabilized-weight IPTW—and showcased consistent directionality across all pre-defined subgroups. To our knowledge, this study supplies the first empirical evidence regarding influenza VE tailored specifically to post-operative children with CHD in China.

The low vaccination coverage observed in our cohort (21.6%) is consistent with previous reports from other regions in China [22,23], suggesting that suboptimal influenza vaccine uptake among children with CHD is a systemic issue rather than a local phenomenon. A recent longitudinal analysis of the SIPIS reported an average annual influenza vaccination coverage of only 2.27% in the Pudong New Area, with rates as low as 0.32% among young adults and only 13.13% receiving three or more doses over a decade [22]. Similarly, a study in Wuxi identified suboptimal uptake of non-National Immunization Program vaccines, including the influenza vaccine, among 4267 children with CHD [23]. This persistently low coverage may be attributed to multiple interrelated factors, including the voluntary and self-paid nature of influenza vaccination, limited awareness among caregivers regarding vaccine benefits, and uncertainty among healthcare providers about vaccine safety in children with chronic conditions. Although expert consensus has clarified that CHD should not be considered a contraindication to vaccination, the translation of this recommendation into clinical practice remains inadequate.

We selected IPTW as the primary analytical approach for deliberate methodological reasons. Observational VE studies are inherently susceptible to confounding by indication [12,24]. IPTW effectively addresses this limitation by constructing a pseudo-population in which treatment assignment is independent of measured covariates, thereby approximating the exchangeability achieved in randomized trials [13,14]. The success of this approach was empirically validated: after weighting, all absolute SMDs fell below 0.10 (range: 0.004–0.043), indicating excellent covariate balance. The doubly robust estimator provided an additional safeguard, yielding consistent estimates if either the propensity score model or the outcome model was correctly specified [15,16]. The near-identical estimates obtained from the doubly robust model (50.9%) and the marginal structural model (50.8%) further support the robustness of our findings and suggest minimal residual confounding.

The VE estimate of 50.9% observed in our study aligns closely with real-world estimates reported for healthy children, which generally range between 50% and 60% depending on annual viral strain matches and circulating subtypes [25]. McLean et al. reported a 71% VE (95% CI: 55–82%) against medically attended influenza during the 2022–23 US season [25], while Doyle et al. cited a 61% VE (95% CI: 49–70%) among children in the 2018–19 season [26]. The consistency of our estimate with these benchmarks delivers an important clinical message: it challenges the prevailing misconception that children with chronic conditions inherently mount insufficient immune responses to vaccination. Our data provide compelling evidence that the influenza vaccine is efficacious—and to a clinically meaningful degree—in post-operative children with CHD.

Subgroup analyses yielded consistently positive VE point estimates across all strata where estimation was feasible. The higher VE observed in females (67.0%) compared to males (49.9%) is biologically plausible, as estrogen has been shown to enhance B-cell differentiation and antibody secretion, leading to stronger vaccine-induced humoral immunity [27,28], whereas testosterone is negatively associated with post-vaccination antibody titers [27]. The substantially attenuated VE in children exposed to passive smoking (2.9%) compared with those unexposed (68.0%) is consistent with established evidence that tobacco-induced impairment damages respiratory epithelial barriers, suppresses local innate immunity, and compromises mucociliary clearance [29]. A recent systematic review and meta-analysis further corroborated that both active and passive smoking significantly depress post-vaccination antibody levels, including for influenza [30]. Among children with straightforward left-to-right shunt lesions, the observed VE of 61.6% is reassuring, given that they represent the majority of clinical cases. In contrast, the wide confidence intervals for the structurally complex “other” lesions category (VE = 73.4%, 95% CI: −53.5% to 95.4%) reflect the extremely small sample size in this subgroup (only six vaccinated patients) and should be interpreted as exploratory rather than indicative of genuine differential effectiveness across lesion types.

Our findings have direct implications for clinical practice and vaccination policy in China. To address the persistently low coverage observed in this high-risk population, we propose several actionable strategies. First, vaccination counseling should be systematically integrated into routine post-operative follow-up appointments, providing an opportunity for healthcare providers to address caregivers’ concerns and actively recommend influenza vaccination. Second, the Shanghai Immunization Information System could be leveraged to identify unvaccinated children with CHD and to facilitate timely reminder and recall systems. Third, targeted educational interventions for both caregivers and healthcare providers are needed to dispel misconceptions about vaccine safety and effectiveness in this population. From a policy perspective, our findings support the consideration of including the influenza vaccine in the National Immunization Program for high-risk pediatric populations or providing subsidized vaccination for children with chronic conditions. Given the substantial morbidity and healthcare utilization associated with influenza in this population, combined with the moderate VE observed in this study, such investment is likely to be cost-effective. Future research should formally evaluate the cost-effectiveness of expanded vaccination strategies and assess their long-term impact on clinical outcomes.

Several limitations of this study should be acknowledged. First, the relatively small sample size (*N* = 194) limited our statistical power for subgroup analyses and prevented formal interaction testing. Second, the reliance on parental recall during telephone interviews may have introduced recall bias, although we mitigated this through calendar-anchoring techniques and by restricting the analysis to medically attended episodes. Third, the absence of laboratory confirmation for ILI diagnoses means that some non-influenza respiratory pathogens were likely captured. This non-differential misclassification would typically bias VE estimates toward the null, suggesting that the true effectiveness against laboratory-confirmed influenza may exceed our estimate of 50.9% [31]. Finally, as a single-center study spanning two specific influenza seasons, the generalizability of our findings to other settings or seasons may be limited. Nevertheless, the high consistency of our VE estimates across four analytical models provides substantial reassurance against residual confounding and strengthens the credibility of our conclusions.

## 5. Conclusions

In conclusion, this retrospective cohort study demonstrates that influenza vaccination yields a 51% reduction in the risk of ILI among post-operative children with CHD in Shanghai, a magnitude of protection mirroring that of healthy pediatric cohorts. This protective efficacy proved remarkably stable across diverse statistical models, including doubly robust IPTW, conventional multivariable regressions, marginal structural models, and stabilized-weight IPTW. By furnishing the initial empirical evidence of influenza VE for this highly specific demographic in China, our findings strongly support clinical guidelines prioritizing their immunization. Conversely, the alarmingly low coverage rate of 21.6% underscores an acute public health deficiency. Decisive, multi-tiered efforts to promote and facilitate vaccination in this immunologically vulnerable subset are absolutely imperative to curtail significant, yet preventable, influenza-related morbidity.

## Figures and Tables

**Table 1:** Baseline characteristics of vaccinated and unvaccinated post-operative CHD children before and after IPTW.

Characteristic	Before IPTW Matching				After IPTW Matching			
	Unvaccinated	Vaccinated	*p*-Value	SMD	Unvaccinated	Vaccinated	*p*-Value	SMD
	*N* = 152	*N* = 42			*N* = 152	*N* = 42		
Age, months, mean ± SD	48.1 ± 20.8	46.1 ± 21.0	0.579	0.098	47.8 ± 1.7	48.7 ± 4.0	0.839	0.041
Sex			0.587	0.126			0.972	0.007
Female	81 (53.3)	25 (59.5)			81 (54.7)	25 (55.1)		
Male	71 (46.7)	17 (40.5)			71 (45.3)	17 (44.9)		
Diagnosis type			0.562	0.146			0.859	0.034
Left-to-right shunt	122 (80.3)	36 (85.7)			122 (81.4)	36 (82.7)		
Other	30 (19.7)	6 (14.3)			30 (18.6)	6 (17.3)		
Passive smoking exposure			0.054	0.424			0.860	0.043
No	119 (78.3)	39 (92.9)			119 (81.4)	39 (83.0)		
Yes	33 (21.7)	3 (7.1)			33 (18.6)	3 (17.0)		
Prior ILI history			1.000	0.033			0.944	0.013
No	136 (89.5)	38 (90.5)			136 (89.6)	38 (89.9)		
Yes	16 (10.5)	4 (9.5)			16 (10.4)	4 (10.1)		
BMI category			1.000	0.090			0.985	0.004
Normal	6 (3.9)	1 (2.4)			6 (3.6)	1 (3.7)		
Abnormal	146 (96.1)	41 (97.6)			146 (96.4)	41 (96.3)		
Any preventive behavior			0.174	0.210			0.860	0.025
No	4 (2.6)	3 (7.1)			4 (3.4)	3 (2.9)		
Yes	148 (97.4)	39 (92.9)			148 (96.6)	39 (97.1)		

SMD < 0.10 was considered indicative of adequate balance between groups after IPTW.

## Data Availability

Restrictions apply to the availability of these data. Clinical and follow-up data used in this study were obtained from Shanghai Children’s Medical Center with permission, and influenza vaccination records were extracted from the Shanghai Immunization Planning Information System (SIPIS). These data are not publicly available due to institutional policies and licensing agreements. They may be accessed from the corresponding author upon reasonable request and with formal approval from the relevant data custodians at Shanghai Children’s Medical Center and SIPIS.

## References

[1] World Health Organization (2022). Vaccines against influenza: WHO position paper—May 2022. Wkly. Epidemiol. Rec..

[2] Iuliano A.D., Roguski K.M., Chang H.H., Muscatello D.J., Palekar R., Tempia S., Cohen C., Gran J.M., Schanzer D., Cowling B.J. (2018). Estimates of global seasonal influenza-associated respiratory mortality: A modelling study. Lancet.

[3] Zhao Q.M., Liu F., Wu L., Ma X.J., Niu C., Huang G.Y. (2019). Prevalence of congenital heart disease at live birth in China. J. Pediatr..

[4] Stephens S.B., Tsang R., Li R., Cazaban-Ganduglia C., Agopian A.J., Morris S.A. (2024). Congenital heart defects and concurrent diagnoses in influenza hospitalization in the Pediatric Health Information System study, 2004–2019. Pediatr. Cardiol..

[5] Ghimire L.V., Chou F.S., Moon-Grady A.J. (2020). Impact of congenital heart disease on outcomes among pediatric patients hospitalized for influenza infection. BMC Pediatr..

[6] Bilgic-Eltan S., Amirov R., Babayeva R., Altunbas M.Y., Karakurt T., Can S., Gungoren E.Y., Bozkurt S., Ozturk N., Catak M.C. (2024). Long-term immunological changes after corrective cardiac surgery. Scand. J. Immunol..

[7] Zhang L., Yang Z., Yin Y., Huang W., Yi T., Ping J., Liu L., Shen P., Sun Y., Lin H. (2024). Using big data to analyze the vaccination status of children with congenital heart disease in Yinzhou District, China. Hum. Vaccines Immunother..

[8] Esposito S., Aurelio C., Cifaldi M., Lazzara A., Viafora F., Principi N. (2026). Prevention of respiratory infections in children with congenital heart disease: Current evidence and clinical strategies. Vaccines.

[9] Chinese National Influenza Surveillance Network Influenza Surveillance Weekly Report, Week 35, 2025. https://www.chinacdc.cn/jksj/jksj04_14249/.

[10] Austin P.C. (2009). Balance diagnostics for comparing the distribution of baseline covariates between treatment groups in propensity-score matched samples. Stat. Med..

[11] Austin P.C., Stuart E.A. (2015). Moving towards best practice when using inverse probability of treatment weighting (IPTW) using the propensity score to estimate causal treatment effects in observational studies. Stat. Med..

[12] Lipsitch M., Tchetgen Tchetgen E., Cohen T. (2010). Negative controls: A tool for detecting confounding and bias in observational studies. Epidemiology.

[13] Cole S.R., Hernán M.A. (2008). Constructing inverse probability weights for marginal structural models. Am. J. Epidemiol..

[14] Robins J.M., Hernán M.A., Brumback B. (2000). Marginal structural models and causal inference in epidemiology. Epidemiology.

[15] Brookhart M.A., Schneeweiss S., Rothman K.J., Glynn R.J., Avorn J., Stürmer T. (2006). Variable selection for propensity score models. Am. J. Epidemiol..

[16] VanderWeele T.J. (2019). Principles of confounder selection. Eur. J. Epidemiol..

[17] Lee B.K., Lessler J., Stuart E.A. (2011). Weight trimming and propensity score weighting. PLoS ONE.

[18] Bang H., Robins J.M. (2005). Doubly robust estimation in missing data and causal inference models. Biometrics.

[19] Funk M.J., Westreich D., Wiesen C., Stürmer T., Brookhart M.A., Davidian M. (2011). Doubly robust estimation of causal effects. Am. J. Epidemiol..

[20] Xu S., Ross C., Raebel M.A., Shetterly S., Blanchette C., Smith D. (2010). Use of stabilized inverse propensity scores as weights to directly estimate relative risk and its confidence intervals. Value Health.

[21] World Health Organization Global Influenza Strategy 2019–2030. https://www.who.int/publications/i/item/9789241515320.

[22] Deng P., Xue C., Yang T., Zheng B., Liu W., Yang L., Fei Y. (2024). Epidemiological analysis of influenza vaccination coverage in Pudong New Area, Shanghai (2013–2023): Implications for influenza vaccination strategies. Hum. Vaccin. Immunother..

[23] Wang L., Yang X., Xiu S., Chen G., Wang X., Shen Y. (2025). Vaccination status in children with congenital heart disease in Wuxi, China: Analysis of retrospective data. Hum. Vaccin. Immunother..

[24] Jackson L.A., Jackson M.L., Nelson J.C., Neuzil K.M., Weiss N.S. (2006). Evidence of bias in estimates of influenza vaccine effectiveness in seniors. Int. J. Epidemiol..

[25] McLean H.Q., Petrie J.G., Hanson K.E., Meece J.K., Rolfes M.A., Sylvester G.C., Neumann G., Kawaoka Y., Belongia E.A. (2023). Interim estimates of 2022–23 seasonal influenza vaccine effectiveness—Wisconsin, October 2022–February 2023. MMWR Morb. Mortal. Wkly. Rep..

[26] Doyle J.D., Chung J.R., Kim S.S., Gaglani M., Raiyani C., Zimmerman R.K., Nowalk M.P., Jackson M.L., A Jackson L., Monto A.S. (2019). Interim estimates of 2018–19 seasonal influenza vaccine effectiveness—United States, February 2019. MMWR Morb. Mortal. Wkly. Rep..

[27] Dhakal S., Park H.S., Seddu K., Lee J.S., Creisher P.S., Seibert B., Davis K.M., Hernandez I.R., Maul R.W., Klein S.L. (2024). Estradiol mediates greater germinal center responses to influenza vaccination in female than male mice. mBio.

[28] Park H.S., Yin A., Zhou W., Wenstedt E.F.E., Jedlicka A., Dziedzic A., Fenstermacher  K.Z.J., Li H., Shea P.J., Lee  J.S. (2025). Sex differences in B cell dynamics after seasonal influenza vaccination are dependent on the age and hormonal profile of vaccinees. medRxiv.

[29] Feldman C., Anderson R. (2013). Cigarette smoking and mechanisms of susceptibility to infections of the respiratory tract and other organ systems. J. Infect..

[30] Valeriani F., Protano C., Pozzoli A., Vitale K., Liguori F., Liguori G., Gallè F. (2024). Does tobacco smoking affect vaccine-induced immune response? A systematic review and meta-analysis. Vaccines.

[31] Eikenboom A.M., Lambregts M.M.C., de Boer M.G.J., le Cessie S. (2024). Influence of initial misdiagnosis on mortality in patients with bacteraemia: Propensity score matching and propensity score weighting analyses. BMC Infect. Dis..

